# Opposite effect of polyamines on *In vitro* gene expression: Enhancement at low concentrations but inhibition at high concentrations

**DOI:** 10.1371/journal.pone.0193595

**Published:** 2018-03-01

**Authors:** Ai Kanemura, Yuko Yoshikawa, Wakao Fukuda, Kanta Tsumoto, Takahiro Kenmotsu, Kenichi Yoshikawa

**Affiliations:** 1 Faculty of Life and Medical Sciences, Doshisha University, Kyoto, Japan; 2 College of Life Sciences, Ritsumeikan University, Shiga, Japan; 3 Department of Chemistry for Materials, Faculty of Engineering, Mie University, Mie, Japan; Emory University School of Medicine, UNITED STATES

## Abstract

**Background:**

Polyamines have various biological functions including marked effects on the structure and function of genomic DNA molecules. Changes in the higher-order structure of DNA caused by polyamines are expected to be closely related to genetic activity. To clarify this issue, we examined the relationship between gene expression and the higher-order structure of DNA under different polyamine concentrations.

**Principal findings:**

We studied the effects of polyamines, spermidine SPD(3+) and spermine SP(4+), on gene expression by a luciferase assay. The results showed that gene expression is increased by ca. 5-fold by the addition of SPD(3+) at 0.3 mM, whereas it is completely inhibited above 2 mM. Similarly, with SP(4+), gene expression is maximized at 0.08 mM and completely inhibited above 0.6 mM. We also performed atomic force microscopy (AFM) observations on DNA under different polyamine concentrations. AFM revealed that a flower-like conformation is generated at polyamine concentrations associated with maximum expression as measured by the luciferase assay. On the other hand, DNA molecules exhibit a folded compact conformation at polyamine concentrations associated with the complete inhibition of expression. Based on these results, we discuss the plausible mechanism of the opposite effect, i.e., enhancement and inhibition, of polyamines on gene expression.

**Conclusion and significance:**

It was found that polyamines exert opposite effect, enhancement and inhibition, on gene expression depending on their concentrations. Such an opposite effect is argued in relation to the conformational change of DNA: enhancement is due to the parallel ordering of DNA segments that is accompanied by a decrease in the negative charge of double-stranded DNA, and inhibition is caused by the compaction of DNA into a tightly packed state with almost perfect charge-neutralization.

## Introduction

**Fig 1:**
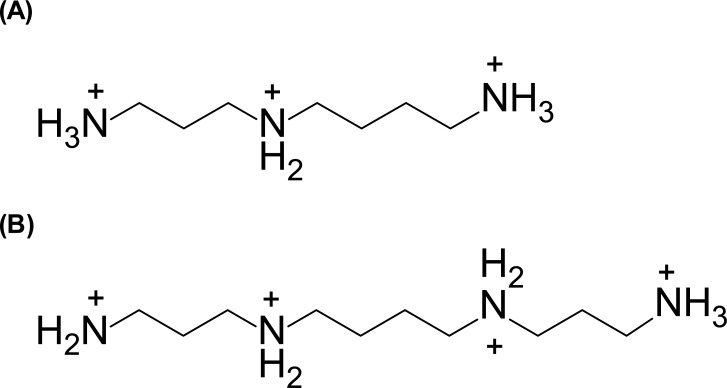
Chemical formulas of (A) spermidine, [SPD(3+)] and (B) spermine, [SP(4+)].

Naturally occurring polyamines such as tri-amine spermidine (SPD(3+)) and tetra-amine spermine (SP(4+)) (Fig 1) are widely distributed in living cells and play essential roles in many biological functions including cell growth and proliferation [1–4]. Under physiological conditions, positively charged polyamines interact with negatively charged macromolecules such as DNA and RNA [5]. Several *in vitro* studies have shown that the binding of polyamines to DNA causes the condensation/compaction of DNA [6–12]. This phenomenon is of great interest in biology and chemistry, since genomic DNA is often found in different degrees of condensation and requires polyamines for the adoption and stabilization of its compact structures [12–14]. Therefore, it is expected that the changes in DNA conformation caused by polyamines could be related to genetic activity. Several studies have examined the relationship between DNA structure and genetic activity [7, 15–17]. Baeza et al. reported that the transcriptional activity of pBR322 DNA in the absence of SPD(3+) was almost the same as that with a concentration of SPD(3+) below the concentration required to induce condensation, whereas the maximum level of transcription was observed with the lowest concentration of SPD(3+) that caused DNA condensation [7]. Tsumoto et al. also studied the effect of SP(4+) on the transcriptional activity of DNA molecules [15–17]. It was found that transcriptional activity of Lambda ZAP II DNA was inhibited in association with the folding transition of DNA to a compact state caused by SP(4+) [15]. However, the detailed morphological features of DNA caused by polyamines and their roles in genetic activity have not yet been fully elucidated. In the present study, we investigated the role of polyamine-induced changes in the structure of DNA on gene expression using atomic force microscopy (AFM) and an *in vitro* luciferase assay.

## Materials and methods

### Materials

Plasmid DNA (Luciferase T7 Control DNA, 4331 bp) containing a T7 RNA polymerase promotor sequence was purchased from Promega (Madison, WI, USA). Spermidine trihydrochloride (SPD(3+)) was purchased from Nacalai Tesque (Kyoto, Japan). Spermine tetrahydrochloride (SP(4+)) was purchased from Wako Pure Chemical Industries (Osaka, Japan). Other chemicals were analytical grade and obtained from commercial sources.

### Luciferase assay for gene expression

Cell-free luciferase assay was performed with a TnT T7 Quick Coupled Transcription/Translation System (Promega) according to the manufacturer’s instructions as follows. Plasmid DNA containing a T7 RNA polymerase promotor sequence was used as the DNA template. The DNA concentration was 0.3 μM in nucleotide units. The reaction mixture was incubated for 90 min at 30°C on a Dry Thermo Unit (TAITEC, Saitama, Japan). Luciferase expression was evaluated following the addition of luciferase substrate (Luciferase Assay Reagent, Promega) by detecting the emission around 565 nm using a luminometer (MICROTEC Co., Chiba, Japan).

### AFM measurements

For AFM imaging using an SPM-9700 (Shimadzu, Kyoto, Japan), 0.3 μM plasmid DNA was dissolved in 10 mM Tris-HCl buffer solution (pH 7.5) with various concentrations of polyamines. The DNA solution was incubated for 10–15 minutes and then transferred onto a freshly cleaved mica surface. The mica surface was not pretreated with any cationic species. After it was allowed to stand for 10 min at room temperature (25°C), the mica was rinsed with water and dried under nitrogen gas. All measurements were performed in air using the tapping mode. The cantilever (OMCL-AC200TS-C2, Olympus, Tokyo, Japan) was 200 μm long with a spring constant of 9–20 N/m. The scanning rate was 0.4 Hz and images were captured using the tapping mode in a 256 × 256 or 512 × 512 pixel format. The obtained images were plane-fitted and flattened by the computer program supplied with the imaging module. An AFM image of DNA at a low concentration of 0.004 mM SPD(3+) or 0.004 mM SP(4+) was used as a control.

## Results

**Fig 2:**
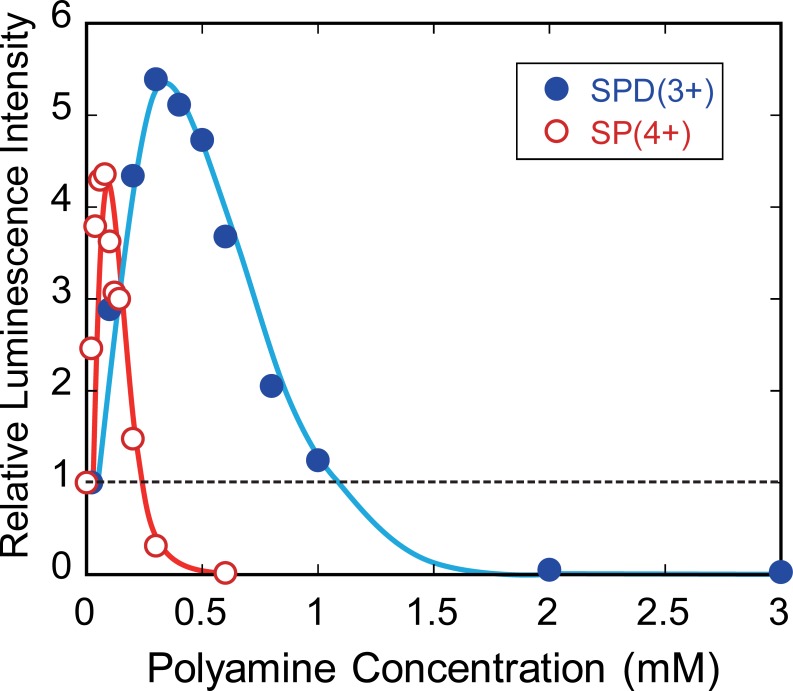
Gene expression efficiency depending on the concentrations of polyamines, SPD(3+) and SP(4+). The longitudinal axis shows the relative emission intensity of luciferin-luciferase reaction, which corresponds to the efficiency of gene expression. DNA concentration was fixed at 0.3 μM.

Fig 2 shows the relative luminescence intensity as a marker of gene expression activity depending on the polyamine concentration. The gene activity increased with an increase in the SPD(3+) concentration and reached a maximum at 0.3 mM. As the SPD(3+) concentration increased further, the activity gradually decreased, and was completely inhibited at 2 mM of SPD(3+). A similar dual effect was observed with SP(4+): the activity reached a maximum at 0.08 mM SP(4+) and complete inhibition was observed above 0.6 mM. Both SPD(3+) and SP(4+) caused a 4- to 5-fold enhancement of gene expression at their respective optimal concentrations, compared to the control. Compared with SP(4+), SPD(3+) required ca. a 4-fold higher concentration for maximal gene expression.

**Fig 3:**
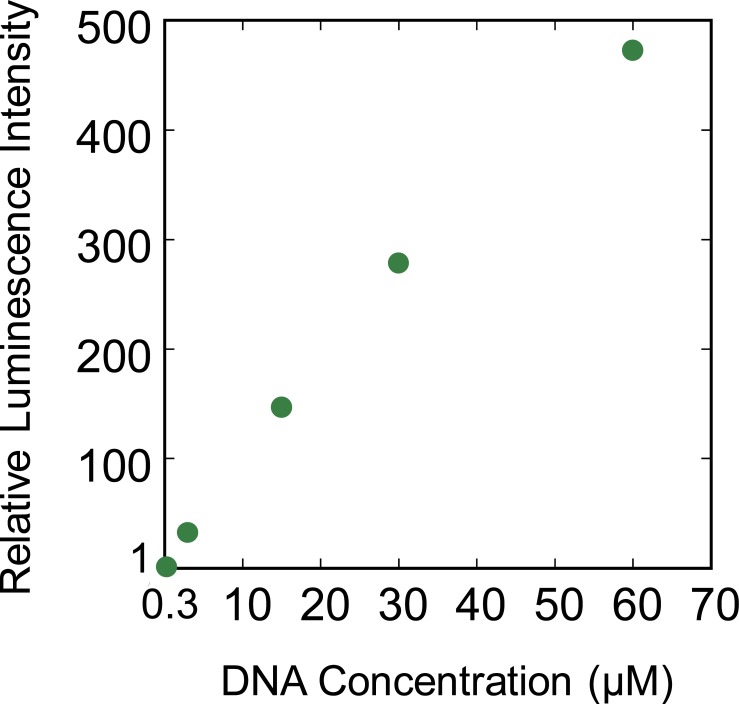
Gene expression efficiency depending on the DNA concentration at a fixed concentration of SPD(3+), 1.5 mM.

To clarify whether complete inhibition of the gene expression is due to the action of polyamines on either enzyme or DNA, we examined the gene expression efficiency with increasing DNA concentration from 0.3 μM to 60 μM at a constant SPD(3+) concentraion (Fig 3). The SPD(3+) concetration was fixed as 1.5 mM at which the gene expression was completely inhibited as shown in Fig 2. As a result, the level of gene expression linearly increased with increasing DNA concentration, suggesting that polyamine inhibits gene expression through the action on DNA not on enzyme.

**Fig 4:**
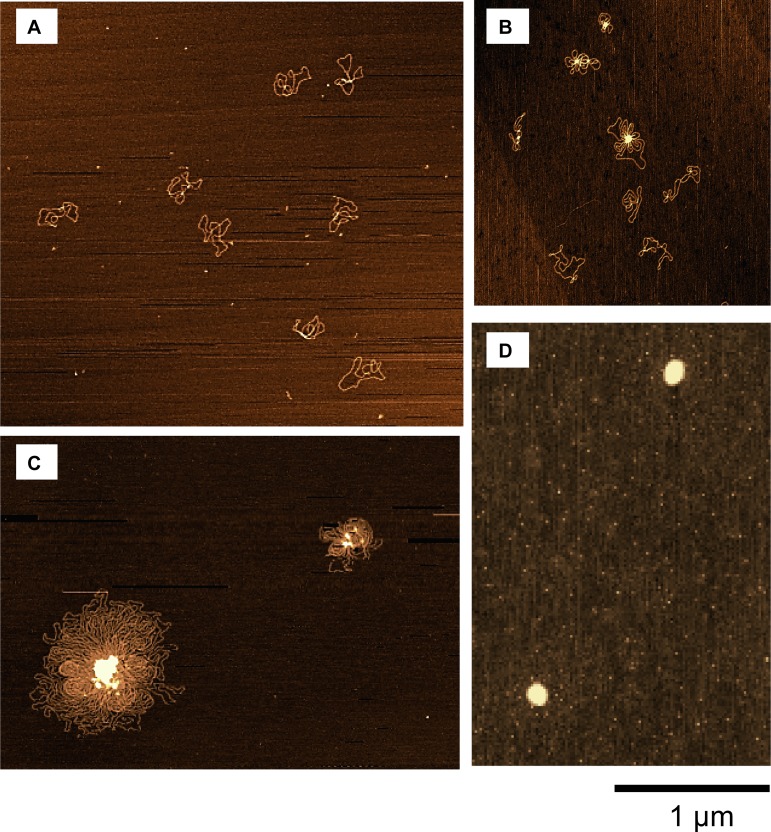
AFM images of DNA conformations at different concentrations of SPD(3+). SPD(3+) concentration is (A) 0.004 mM (control), (B) 0.1 mM, (C) 0.3 mM, and (D) 2 mM.

**Fig 5:**
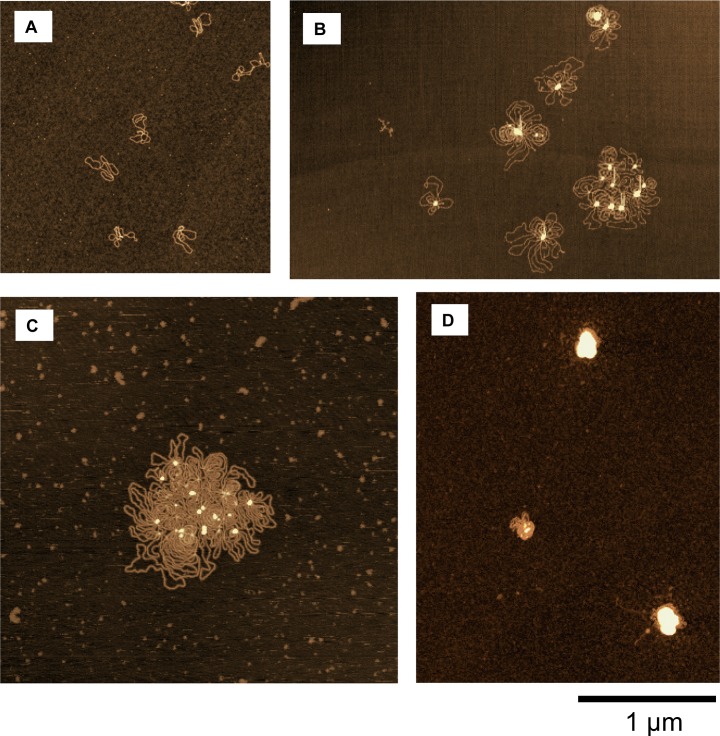
AFM images of DNA conformations at different concentrations of SP(4+). SP(4+) concentration is (A) 0.004 mM (control), (B) 0.007 mM, (C) 0.06 mM, and (D) 0.6 mM.

To shed light on the relationship between gene activity and the conformation of DNA, we observed the DNA conformation under treatment with polyamines by AFM. Fig 4 exemplifies the conformation of circular DNA molecules deposited onto a mica surface under different concentrations of SPD(3+). At 0.004 mM SPD(3+), DNA molecules were separately dispersed in a relaxed state on the mica surface (Fig 4A). Dispersed individual DNA molecules with the tendency to form intrachain loops were observed with an increase of SPD(3+) to 0.1 mM (Fig 4B). At 0.3 mM, a flower-like complex was formed accompanied by the assembly formation with plural number of DNA moelcules (Fig 4C). At 2 mM, a tightly packed conformation appeared (Fig 4D). A similar conformational change was observed with an increase in SP(4+) (Fig 5). With the increase of SP(4+) concentration, flower-like patterns appreaed accompaning the formation of loops (Fig 5B and 5C). At 0.6 mM, DNA molecules exhibited tightly packed conformation(Fig 5D).

In our observation, flower-like pattern was seen for an assemblies with multiple DNA molecules (4.3 kbp) as in Figs 4C and 5C, revealing the parallel orientation of neighboring DNA segments. Similar flower-like conformation as an early intermediate in SPD(3+)-induced condensation was reported for 3 kbp DNA through AFM observation by Fang and Hoh [10]. Here, it is noted that AFM provides detailed morphological change of DNA molecules for the specimens attached/adsorbed on a solid substrate and, thus, we should take care the difference from the conformation in 3-dimensional solution environment. Bearing in mind the possible modification on the 2-dimensional DNA conformation adsorbed on a solid substrate as observed by AFM, in the following we will discuss the structural effect of DNA on the activity of gene expression at different polyamine concentrations.

## Discussion

Our experimental observations clearly indicate that polyamines have opposite effect on gene expression under the present condition, i.e., enhancement and inhibition, at lower and higher concentrations, respectively. AFM observation showed the generation of a flower-like conformation at polyamine concentrations corresponding to the highest level of gene expression. The generation of a flower-like pattern as exemplified in the AFM observations (Figs 4C and 5C) is expected to be associated with the tendency for DNA segments to prefer parallel ordering [18, 19]. We will now discuss this specific effect of polyamines in terms of counter ion condensation around double-stranded DNA molecules. It has been well established that strongly-charged polyelectrolytes exhibit a strong spatial correlation among ions, where the usual Poisson-Boltzmann approximation breaks down; i.e., the degree of dissociation, *η*, of phosphate groups is significantly reduced due to counter ion condensation, under the condition that the only counter ion is a monovalent cationic ion [20–22].

$$\eta =\frac{d}{l_{B}}$$

where lB=q2εkBT is the Bjerrum length; *q*: unit charge, *ε*: dielectric constant (78 at 25°C), *k_B_*: Boltzmann constant, and *T*: absolute temperature. Thus, in usual aqueous solution, $l_{B}$ is 0.7 nm at 25°C. *d* is the distance between charged moieties. For B-DNA, the distance between neighboring base pairs is 0.34 nm, and each base pair contains two phosphate groups. If we simply divide 0.34 nm by 2, we obtain *d* ≅ 0.17 nm. Thus, the degree of dissociation is given as *η* ≅ 0.24. This means that ca. 76% of the intrinsic negative charges or phosphate groups are neutralized because they attract cationic counter ions from the surrounding solution, and only 24% remain dissociated to provide a negative charge on DNA. By introducing a parameter *θ*_1_ to express the degree of charge neutralization due to counter ion condensation in a solution with only monovalent cations, we may write *θ*_1_ ≅ 0.76. For a solution with *z*-valent cationic counter ions, the degree of charge neutralization is generally considered to be *θ*_*Z*_ = 1 − *η*/*z*. Thus, for solutions with di-valent, tri-valent and tetra-valent counter cations, *θ*_2_ ≅ 0.88, *θ*_3_ ≅ 0.92, and *θ*_4_ ≅ 0.94. In the actual solution conditions, polyamines, as multivalent cationic species, are present together with monovalent cations. Thus, with an increase in the polyamine concentration, it is expected that the degree of counter ion condensation or charge neutralization will gradually increase [23, 24]. For example, when SPD(3+) is added to a solution in the presence of a certain amount of monovalent cations in the buffer, the degree of SPD(3+) cations located on double-stranded DNA will increase; the degree of charge neutralization gradually increases from 0.76 to 0.92 with an increase in SPD(3+) until the critical concentration to cause compaction. Thus, the effective negative charge of DNA molecules gradually decreases to ~8% of the original value (in terms of the number of phosphate groups) with an increase in the SPD(3+) concentration. Similarly, in the case of SP(4+), the negative charge decreases to ~6% of the original value. Thus, DNA molecules in solutions of around 0.3 mM SPD(3+) and 0.08 mM SP(4+) are considered to exhibit negative charges of about 8% and 6%, respectively, of the original values (in terms of the number of phosphate groups), where the DNA conformation is slightly shrunken compared to the conformation in the absence of polyamine. Under these conditions, DNA segments prefer a parallel alignment because of the self-avoiding effect of the segments in a slightly shrunken DNA chain, which is similar to the mechanism of stabilization with liquid crystalline ordering [18, 19]. Thus, parallel ordering among the stiff segments with weak repulsive interaction through the surviving negative charge provides greater stability than a random orientation, because of the large freedom of fluctuation in the parallel alignment, i.e., there is greater entropy for parallel ordering than for a random orientation among the DNA segments. With a further increase in the levels of polyamines, DNA tends to fold into a compact state. Such a folding transition is markedly discrete in giant DNA molecules above a size of several tens of kbp [9, 25–27]. The resulting compact state is almost neutral with respect to charge density due to the enhancement of multivalent cation binding accompanied by the folding transition [28, 29]. In *in vitro* experiments, a complete inhibition of transcriptional activity was observed when SP(4+) was added to the transcriptional solution above a threshold concentration, corresponding to a condition that causes the folding transition to a compact state [15–17]. On the other hand, for the parallel alignment of segments with a surviving negative charge of 6–8%, i.e., in the flower-like conformation, each DNA segment tends to avoid crossing or condensation with other segments.

**Fig 6:**
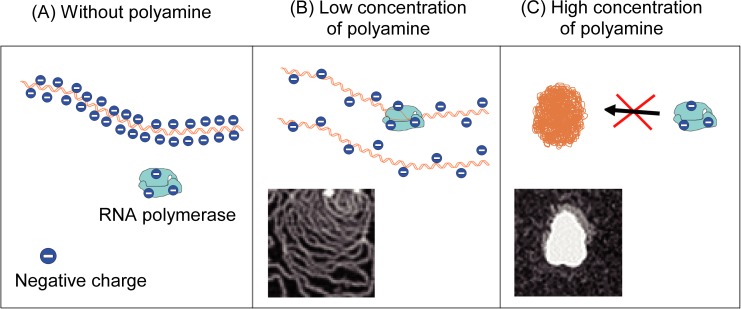
Schematic representation of the nature of the interaction of polyamine with DNA and also with RNA polymerase depending on the polyamine concentration. (A): elongated DNA without polyamine, (B): parallel alignment of DNA segments with the inset of the AFM image of Fig 5(C), (C): compact DNA with the inset of AFM of Fig 5(D).

The schematic drawings of DNA conformation shown in Fig 6 illustrate the change of negative charge along at different polyamine concentrations. It is known that RNA polymerase is negatively charged in a usual aqueous environment [30–32]. Thus, it is expected that polyamines will generate favorable conditions for RNA polymerase to access DNA segments with a reduced negative charge. In addition, substrates for the transcriptional reaction, i.e., NTPs, are also negatively charged. Polyamine may decrease the negative charge not only on DNA and RNA polymerase but also on these substrates, which causes favorable solution conditions to accelerate the transcriptional and translational reactions. Although, the mechanism by which polyamines promote gene expression is not yet fully clarified, it is expected that polyamines can have favorable effects in a similar scenario with a decrease in repulsive interactions among negatively charged species. It may be useful to make clear the mechanism why polyamines concern with a wide variety of biological functions [33, 34] through ‘nonspecific interaction’ under the proposed scenario.

The living organisms is a highly complex system consisting of a number of elements. Our experiments were conducted in a simplified model system to focus on the action of polyamines on DNA. Further studies in the nonspecific but significant effect of polyamines on DNA and other negatively-charged biomolecules by taking into account the influence of cationic species [35, 36] such as K^+^ and Mg^2+^ would provide useful insight regarding mechanisms of living matters as autonomous self-control system.

## Abbreviations

SPD(3+): spermidine
SP(4+): spermine
AFM: atomic force microscopy
Bp: base pairs
